# Efficient Scientific Self-Correction in Times of Crisis

**DOI:** 10.1007/978-3-030-65355-2_23

**Published:** 2021-03-20

**Authors:** Michèle Nuijten

**Affiliations:** 1 Tilburg University, Tilburg, The Netherlands; 2 Tilburg University, Tilburg, The Netherlands; 3 Tilburg University, Tilburg, The Netherlands; 4 Tilburg University, Tilburg, The Netherlands; Department of Methodology and Statistics, Tilburg School of Social and Behavioral Sciences, Tilburg University, Tilburg, The Netherlands

## Abstract

Science has been invaluable in combating the COVID-19 pandemic and its consequences. However, science is not flawless: especially research that is performed and written up under high time pressure may be susceptible to errors. Luckily, one of the core principles of science is its ability to self-correct. Traditionally, scientific self-correction is achieved through replication, but this takes time and resources; both of which are scarce. In this chapter, I argue for an additional, more efficient self-correction mechanism: analytical reproducibility checks.

When the COVID-19 pandemic hit in early 2020, the scientific community was quick to respond. Within 4 months after the first reported COVID-19 case, over 13,000 papers related to COVID-19 were published in scientific journals. On top of that, over 7000 preprints (self-published PDFs) were posted online (see Fig. 23.1; Fraser et al. 2020; Fraser and Kramer 2020).

**Fig. 23.1:**
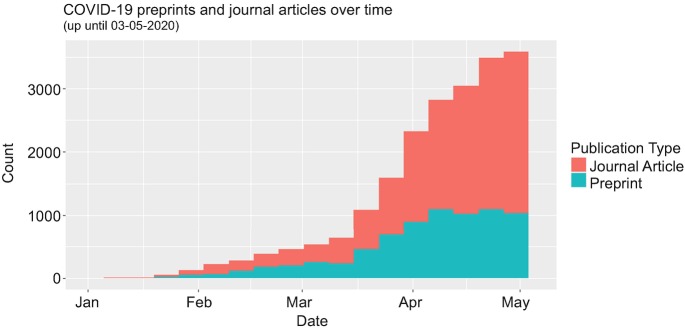
Number of COVID-19 preprints and journal articles over time (data source: Fraser and Kramer (2020))

It is encouraging to see the speed with which the scientific community has responded to the pandemic. Perhaps even more encouraging is that science has played such an important role in shaping policies and interventions against COVID-19 and its consequences. If there was ever a time in which the importance of science to society was highlighted, it is now.

However, it is important to keep in mind that science is a human endeavor and, therefore, not flawless. Scientific publications, both preprints and peer-reviewed articles, can be affected by errors and bias.

Already, we have seen some high-profile cases of flawed papers in the COVID-19 literature. For example, a paper published in the prestigious journal *The Lancet* reported that the antimalarial drug hydroxychloroquine could be dangerous to people with COVID-19. This finding brought an abrupt halt to multiple clinical trials in which this drug was tested as a potential treatment of COVID-19. Almost immediately after publication, the scientific community noted that it was highly unlikely that such a large and detailed database on COVID-19 was collected in such a short time and started questioning the validity of the findings. When the authors could not verify the data, the article was retracted, along with another high-profile article by the same authors that was based on the same dataset (for more details, see e.g., Davey 2020; Ledford and Van Noorden 2020; Rabin and Gabler 2020). These retractions raised questions about the potential risks of conducting research under so much time pressure. The global crisis may have prompted researchers to cut corners in data collection and analysis in order to get their studies out there as soon as possible. Lower levels of scrutiny are never desirable but may be particularly problematic when scientific findings are communicated and sometimes even implemented before formal peer review has taken place. To quickly separate the weed from the chaff in the COVID-19 literature, we need an efficient correction mechanism.

## Scientific Self-Correction

Science is often said to be self-correcting, reflecting the idea that science is an iterative process that will lead us to “the truth” step by step by constantly updating information. Self-correction should weed out findings that turn out to be flukes or even errors. But self-correction does not happen magically overnight. Someone has to actively correct the scientific record for scientific “self-correction” to take place (see also Vazire 2019).

The main self-correction mechanism is replication. In a replication study, researchers collect and analyze new data while closely following the methodology of the original study. If the replication study shows the same results as the original study, the results are corroborated. However, if the replication study shows different results, it may undermine the trust in the original finding. Especially if a string of replication studies keeps showing different results than the original study, the original result is eventually discarded in favor of the replications’ results.

A downside of replication studies is that they can take a lot of money and time, both of which are scarce. Especially during times of crisis, such as the current COVID-19 pandemic when we need fast answers to our questions, it is important to have efficient correction mechanisms at hand.

## Reproducibility Checks as an Efficient Self-Correction Mechanism

I would like to add an additional, more efficient tool to the self-correction toolbox: analytical reproducibility checks, or simply reproducibility checks. A paper is successfully reproduced when reanalysis of the original data, following the original strategy, produces the same results as reported in the paper. Note that, as opposed to replication, reproducibility checks do not involve collecting new data. This makes reproducibility checks much quicker and cheaper than a replication.

It may seem self-evident that reanalyzing the same data following the same strategy as the original authors leads to the same results. Unfortunately, this is often not the case. Not only errors in the data cleaning and typos in reporting results but also lack of clarity in describing analyses or unavailable data can all result in findings that are not reproducible (Hardwicke et al. 2018; Ioannidis et al. 2009; Nuijten et al. 2016; Stodden et al. 2018).

Reproducibility is a minimum standard for research quality (Nuijten et al. 2018; Peng 2011). If it is unclear how the data led to the reported findings, these findings cannot be substantively interpreted. The importance of reproducibility for interpretation became clear in the hydroxychloroquine case described above, where the paper was retracted because the findings were not reproducible: neither the readers nor the authors themselves were able to reproduce the reported results based on the data.

Many conclusions in the COVID-19 literature are based on statistical analyses. Think about estimates of the mortality rate, assessments of the accuracy of COVID-19 test kits, or tests whether a treatment is effective by comparing means in experimental and control conditions. In such cases, reproducibility checks may be an efficient tool to quickly verify reported results.

## Detecting Reproducibility Problems

Reproducibility checks can be done at different effort and complexity levels. A reproducibility check could consist of an in-depth reanalysis of the original data, but some reproducibility problems can be spotted without access to raw data. The latter are so-called “statistical reporting inconsistencies” that can be detected in the paper itself. Such an inconsistency arises when the numbers belonging to a set do not match.

Consider the following fictional example. Say that a paper states that “7% of the patients with Covid-19 died in hospital (5/100).” Purely based on the reported results, it can be concluded that the numbers are not internally consistent: 5 out of 100 patients is 5%, not 7%. At this point, it is unclear which of the reported numbers is incorrect. What is clear, however, is that the result in its current form is not reproducible and, therefore, not reliable: even without reanalyzing the underlying data, we can conclude that it is impossible to arrive at this combination of numbers.

Reporting inconsistencies can occur in a wide variety of statistics. For example, the reported accuracy of a test kit should be consistent with the reported true positive, true negative, false positive, and false negative rates. Similarly, the reported total sample size should match subgroup sizes, odds ratios should match raw frequencies, reported *p*-values of statistical hypothesis tests should match their test statistics and degrees of freedom, etcetera.

Screening a paper for reporting inconsistencies is an efficient way to detect reproducibility problems: it can be done quickly and you have immediate, objective feedback about the trustworthiness of a particular result. A next step could be a full reanalysis of the original data to see if the same numbers can be reproduced. Such a reanalysis could possibly be extended by sensitivity analyses: do the results still hold up under different (justifiable) analytical choices? For example, what happens to the effect when one extreme observation is removed? Or when the analysis is redone without an arbitrary covariate?

I would argue that if any of the steps above do not hold, the result is not robust. Either it is unclear how the data led to the reported results—in which case the results cannot be meaningfully interpreted—or the results only hold under a highly specific set of analytical choices. In such cases, we may not need to perform a replication study in a new sample to determine whether or not to trust the study.

## Closing Remarks

Especially in COVID-19 research, where new scientific findings are sometimes immediately implemented, we need quick ways to determine whether the reported findings are trustworthy. Relying on “traditional” scientific self-correction in the form of replication studies in new samples may not be sufficient: replication takes a lot of time and—maybe more importantly—if the results in the original study are erroneous, it is not possible to meaningfully compare them to the replication results. Systematic reproducibility checks could be an efficient way to spot errors and speed up scientific self-correction in the COVID-19 literature.

The current pandemic provides an incentive to reassess the way science progresses. It highlights the risks of “rushed” science and emphasizes the need for efficient robustness checks. But efficient robustness checks are not only relevant in times of crisis: society progresses faster than ever and science needs to work hard to keep up. We can take this opportunity to develop new habits in the way we conduct science by systematically assessing the reproducibility of results, screening papers for reporting inconsistencies, reanalyzing data, and performing sensitivity checks. Additionally, we can use this logic not only to assess, but also to improve the robustness of our results by fully reporting our statistical results, sharing our data and analysis scripts, and reporting results of alternative analysis strategies.

By recognizing the importance of the link between the data and the reported results—the importance of reproducibility—we can improve scientific self-correction and scientific progress in times of crisis and beyond.
